# Mutations of *C-Reactive Protein* (*CRP*) -286 SNP, *APC* and *p53* in Colorectal Cancer: Implication for a CRP-Wnt Crosstalk

**DOI:** 10.1371/journal.pone.0102418

**Published:** 2014-07-15

**Authors:** Hai-Xiang Su, Hai-Hong Zhou, Ming-Yu Wang, Jin Cheng, Shi-Chao Zhang, Feng Hui, Xue-Zhong Chen, Shan-Hui Liu, Qin-Jiang Liu, Zi-Jiang Zhu, Qing-Rong Hu, Yi Wu, Shang-Rong Ji

**Affiliations:** 1 Gansu Provincial Academic Institute for Medical Research, Lanzhou, P.R. China; 2 The Gansu Provincial Tumor Hospital, Lanzhou, P.R. China; 3 MOE Key Laboratory of Cell Activities and Stress Adaptations, School of Life Sciences, Lanzhou University, Lanzhou, P.R. China; 4 Key Laboratory of Preclinical Study for New Drugs of Gansu Province, Lanzhou University, Lanzhou, P.R. China; Morehouse School of Medicine, United States of America

## Abstract

C-reactive protein (CRP) is an established marker of inflammation with pattern-recognition receptor-like activities. Despite the close association of the serum level of CRP with the risk and prognosis of several types of cancer, it remains elusive whether CRP contributes directly to tumorigenesis or just represents a bystander marker. We have recently identified recurrent mutations at the SNP position -286 (rs3091244) in the promoter of *CRP* gene in several tumor types, instead suggesting that locally produced CRP is a potential driver of tumorigenesis. However, it is unknown whether the -286 site is the sole SNP position of *CRP* gene targeted for mutation and whether there is any association between *CRP* SNP mutations and other frequently mutated genes in tumors. Herein, we have examined the genotypes of three common *CRP* non-coding SNPs (rs7553007, rs1205, rs3093077) in tumor/normal sample pairs of 5 cancer types (n = 141). No recurrent somatic mutations are found at these SNP positions, indicating that the -286 SNP mutations are preferentially selected during the development of cancer. Further analysis reveals that the -286 SNP mutations of *CRP* tend to co-occur with mutated *APC* particularly in rectal cancer (*p* = 0.04; n = 67). By contrast, mutations of *CRP* and *p53* or *K-ras* appear to be unrelated. There results thus underscore the functional importance of the -286 mutation of *CRP* in tumorigenesis and imply an interaction between CRP and Wnt signaling pathway.

## Introduction

Inflammation is essential for the development of cancer [1], [2]. As a major human acute phase reactant, C-reactive protein (CRP) is widely used as a non-specific marker of inflammation [3], [4]. However, accumulating evidence has revealed a close association between the serum level of CRP and the risk and prognosis of cancer [5]. Given the presumed functions of CRP in host defense and innate immunity [6], [7], it is plausible that CRP may play a direct role in tumorigenesis. Indeed, CRP has been reported to prevent the apoptosis of myeloma cells [8] and to facilitate the invasiveness of breast cancer cells [9]. Moreover, CRP may contribute to the establishment of a favorable tumor microenvironment by promoting angiogenesis [10], by inhibiting the destructive activation of complement [11], [12], and by inducing proinflammatory cytokines from immune and endothelial cells [3], [13], [14].

On the other hand, single nucleotide polymorphisms (SNPs) that associate with genetically elevated concentrations of CRP do not confer an increased cancer risk to the general population [15]. This suggests that circulating CRP is not causally involved in tumorigenesis. Intriguingly, in contrast to the aforementioned pro-cancer activities, early studies have also documented anti-cancer actions of CRP through activation of macrophage/monocyte [16]–[18]. Consequently, it has been difficult to define whether CRP is solely a passive marker or an active player in cancer, or to dissect the exact contribution of CRP in tumorigenesis.

Serum CRP is produced by hepatocytes of the liver; however, accumulating evidence also reveals a local production of CRP by extra-hepatic cells [3], [19]. Interestingly, we have recently found that the promoter of *CRP* is specifically mutated at the SNP position (rs3091244) 286 bp upstream the transcription start site in 109 out of 453 tumor samples but not in the matched normal controls [19]. These mutations are associated with enhanced local *CRP* induction in tumors likely via disruption of the conserved CpG methylation motif. Moreover, most of the cancer types examined harbor the -286 mutation and the fraction of the mutated allele is high (0.487, 95% CI: 0.477–0.517). These findings thus support the role of CRP produced *in situ* as a potential cancer driver that is probably involved in general mechanisms favoring tumorigenesis [19].

Besides the -286 SNP, there are several additional common non-coding SNPs that significantly affect the baseline levels of serum CRP. The representatives include rs7553007, rs1205, and rs3093077 [15], [20], [21]. It is therefore of interest whether these SNP sites are also targeted for mutation in tumors. We show here by genotyping of 141 tumor/normal sample pairs that no recurrent mutations occur at the 3 *CRP* SNP sites, thus highlighting that the -286 mutations are highly specific to tumorigenesis. We further examined whether there is any correlation between the -286 mutations of *CRP* and other frequently mutated genes in tumors. The identified association between the -286 and *APC* mutations implies an interaction of CRP with Wnt signaling.

## Materials and Methods

Frozen tumor/normal tissue sample pairs were obtained from the tissue bank of Gansu Provincial Tumor Hospital. Genomic DNA was isolated from tissues or blood samples using DNAiso Reagent or Blood Genome DNA Extraction Kit (Takara) according to the manufacturer’s instructions. For the identification of gene mutations, genomic DNA was amplified with specific primers (human *CRP*: forward: 5′-AGGGGGGAGGGATAGCATTAGAA-3′; reverse: 5′-CGTCCTGCTGCCAGTGATACAAG-3′; human *p53*: forward: 5′-CTGTCCCTTCCCAGAAAACCT-3′; reverse: 5′-CCTGGGCATCCTTGAGTTC-3′; human *APC*: forward: 5′-TAATACCCTGCAAATAGCAGAAATA-3′; reverse: 5′-GTGGCAAAATGTAATAAAGTATCAG-3′; human *K-ras*: forward: 5′-ATGACTGAATATAAACTTGTGGTA-3′; reverse: 5′-CAACACCCTGTCTTGTCTT-3′) followed by sequencing. Genotyping of 24 SNPs (rs7553007, rs1205, rs3093077, rs4073, rs1143627, rs720816, rs723504, rs1876054, rs746961, rs2371923, rs487616, rs953183, rs400328, rs950487, rs759394, rs726402, rs1568645, rs929689, rs2254896, rs1951096, rs1843026, rs1543193, rs718015, rs16091) was performed by the mass spectrometry-based Sequenom service (Genergy Biotechnology, Shanghai, China). Written informed consent was obtained from patients. All patients are Chinese. The study was approved by the Ethic Committee of the Gansu Provincial Tumor Hospital.

## Results

### No recurrent somatic mutations occur at 3 common *CRP* SNP sites in tumors

**Figure 1:**
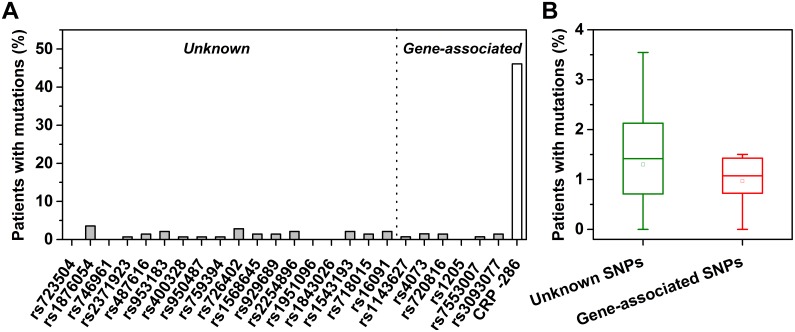
Percentage of patients with somatic mutations at the indicated SNP sites in tumors. 3 *CRP* SNPs (rs7553007, rs1205, and rs3093077) and 21 additional SNPs of 141 tumor/normal sample pairs were genotyped by Sequenom. These samples were collected from 37 gastric, 12 lung, 27 esophagus, 24 colon and 41 rectal cancer patients. (A) The mutation frequencies at each SNP sites. None of these sites is recurrently mutated in tumors. The frequency of the *CRP*-286 SNP (rs3091244) mutation in these samples is shown for comparison. (B) The pooled mutation frequencies of SNPs with or without associated genes. Gene-associated SNP sites tend to exhibit lower mutation frequencies albeit without reaching statistical significance (two sample *t* test, two-tailed, *p* = 0.47).

To see whether other non-coding SNP sites of *CRP* are mutated in tumors, we determined the genotypes of 3 *CRP* common SNPs (rs7553007, rs1205, and rs3093077) together with 21 additional SNPs in 141 tumor/normal sample pairs of 5 cancer types, *i.e.* gastric, lung, esophagus, colon and rectal cancers. The frequencies of alleles associated with lower CRP levels are 48.9% for A allele of rs7553007, 47.9% for T allele of rs1205, and 79.1% for T allele of rs3093077 in normal samples (Table 1), thus providing sufficient sample sizes for detection of recurrent mutations. Therefore, we identified only 1 case of G>A mutation at rs7553007, 0 case of mutation at rs1205, and 2 cases of G>T mutations at rs3093077 in the matched tumor samples. Such a low incidence of somatic mutation was also found for 21 other examined non-coding SNP sites distributed across 9 different chromosomes (Figure 1). These indicate that, in contrast to the highly recurrent *CRP*-286 SNP (rs3091244) mutations [19], the 3 *CRP* SNP sites assayed herein are only randomly mutated in tumors at the background mutation frequency.

**Table 1:** Clinicopathologic features of 141 cancer patients whose tumor/normal sample pairs were genotyped.

			Allele frequencies, %			
		Number of patients (%)	rs3091244	rs7553007	rs1205	rs3093077
**N**		141				
**Age**	<58 y	66 (47)				
	≥58 y	75 (53)				
**Gender**	Female	38 (27)				
	Male	103 (73)	C: 77.0%	G: 51.1%	C: 52.1%	T: 79.1%
**Tumor Stage**	0–2	57 (40)	A: 14.5%	A: 48.9%	T: 47.9%	G: 20.9%
	3–4	84 (60)	T: 8.5%			
**Chemotherapy status**	Naïve	113 (80)				
	Prior treatment	28 (20)				

According to the genotyping results, the mutation frequencies of SNP sites with and without associated genes are 0.97% (95% CI: 0.35–1.59%) and 1.30% (95% CI: 0.78–1.82%), respectively. Although not statistically significant, this suggests that gene-associated SNP sites tend to be less prone to random mutation than those with unknown association, possibly due to constraints that limit damages to genomic loci with functional importance. Of the gene-associated SNPs, rs1143627 and rs4073 are two promoter SNPs that locate at 31 and 199 bp upstream of the transcription start sites of *IL-1β* and *IL-8*, respectively. Their low mutation frequencies (0.7–1.4%) argue that the promoter localization *per se* is not likely the cause of somatic hypermutation at the *CRP*-286 SNP -site in tumors; rather, the high incidence of the -286 mutation would be the result of functional consequences related to the enhanced induction of *CRP*, which may confer host cell clones sufficient advantage to survive and expand in the development of cancer.

### *CRP*-286 SNP mutation is associated with mutated *APC* in rectal cancer

**Figure 2:**
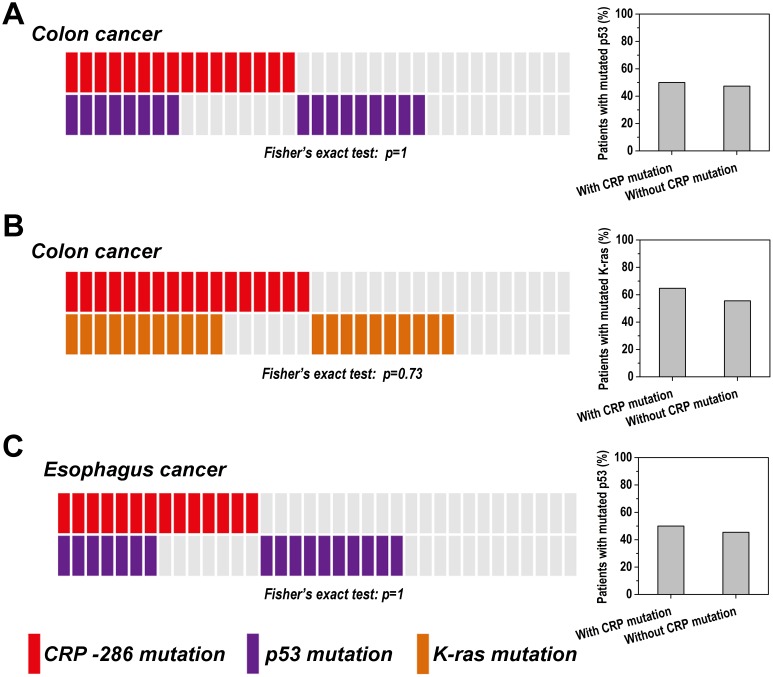
The distribution of somatic mutations of *CRP*-286 SNP, *p53* and *K-ras* in tumors. The *CRP*-286 SNP mutations show no association with mutated *p53* or *K-ras* in colon (A-B) or esophagus cancers (C) (Fisher’s exact test, two-tailed). Each rectangle represents one tumor sample with grey color denotes wild type status. The bar graphs on the right show the percentages of patients carrying the indicated mutations in two patient groups with or without CRP-286 mutations.

The *CRP*-286 SNP mutation is most prevalent in colon cancers [19], in which *p53*, *K-ras* and *APC* are among the most frequently mutated genes that promote tumorigenesis via distinct mechanisms [22]–[24]. We thus sought to examine whether there is any association between these mutation events. Mutated *p53*, *K-ras* and *APC* were identified by sequencing of their respective hotspot mutation regions, *i.e.* 301–1044 of *p53*, 24–442 of *K-ras*, and 3922–4453 of *APC* in cDNA sequence ranges, according to the statistics of the COSMIC database. Despite their high incidences (about 50%), the *CRP-*286 SNP mutation shows no apparent association with mutated *p53* (n = 35; Table 2 and Fig. 2A) or *K-ras* (n = 35; Table 3 and Fig. 2B). The lack of association between *p53* and the *CRP*-286 SNP mutations was also confirmed in esophagus cancer (n = 36; Table 4 and Fig. 2C), wherein *p53* represents the most frequently mutated gene.

**Table 2:** Clinicopathologic features of 35 colon cancer patients whose tumor/normal sample pairs were examined for *p53* mutations.

		Number of Patients (%)	Number of patients with*CRP*-286 mutation (%)	*p* *	Number of patients with*p53* mutation (%)	*p* *
**N**		35				
**Age**	<57 y	17 (49)	9 (56)	0.51	9 (53)	0.74
	≥57 y	18 (51)	7 (44)		8 (47)	
**Gender**	Female	13 (37)	6 (37.5)	1	6 (35)	1
	Male	22 (63)	10 (62.5)		11 (65)	
**Tumor Stage**	0–2	19 (54)	9 (56)	1	8 (47)	0.51
	3–4	16 (46)	7 (44)		9 (53)	
**Chemotherapy** **status**	Naïve	30 (86)	13 (81)	0.64	15 (88)	1
	Prior treatment	5 (14)	3 (19)		2 (12)	

*Fisher’s exact test, two-tailed.

**Table 3:** Clinicopathologic features of 35 colon cancer patients whose tumor/normal sample pairs were examined for *K-ras* mutations.

		Number of Patients (%)	Number of patients with*CRP*-286 mutation (%)	*p* *	Number of patients with*K-ras* mutation (%)	*p* *
**N**		35				
**Age**	<56 y	17 (49)	10 (59)	0.32	9 (43)	0.50
	≥56 y	18 (51)	7 (41)		12 (57)	
**Gender**	Female	12 (34)	6 (35)	1	9 (43)	0.28
	Male	23 (66)	11 (65)		12 (57)	
**Tumor Stage**	0–2	20 (57)	10 (59)	1	13 (62)	0.51
	3–4	15 (43)	7 (41)		8 (38)	
**Chemotherapy status**	Naïve	29 (83)	13 (76)	0.40	20 (95)	0.03
	Prior treatment	6 (17)	4 (24)		1 (5)	

*Fisher’s exact test, two-tailed.

**Table 4:** Clinicopathologic features of 36 esophagus cancer patients whose tumor/normal sample pairs were examined for *p53* mutations.

		Number ofPatients (%)	Number of patientswith*CRP*-286mutation (%)	*p* *	Number of patientswith*p53* mutation(%)	*p* *
**N**		36				
**Age**	<61 y	16 (44)	8 (57)	0.31	10 (59)	0.18
	≥61 y	20 (56)	6 (43)		7 (41)	
**Gender**	Female	4 (11)	2 (14)	0.63	3 (18)	0.33
	Male	32 (89)	12 (86)		14 (82)	
**Tumor Stage**	0–2	21 (58)	5 (36)	0.04	10 (59)	1
	3–4	15 (42)	9 (64)		7 (41)	
**Chemotherapy status**	Naïve	32 (89)	10 (71)	0.02	16 (94)	0.61
	Prior treatment	4 (11)	4 (29)		1 (6)	

*Fisher’s exact test, two-tailed.

**Figure 3:**
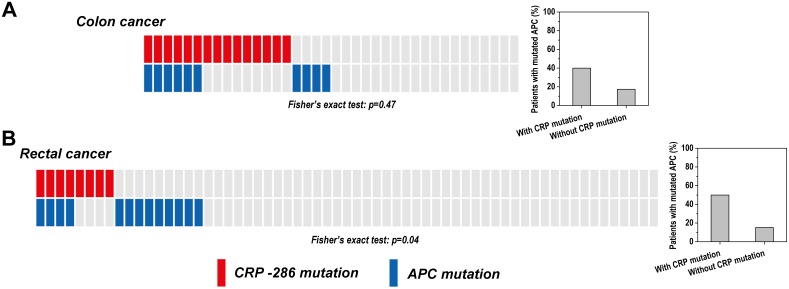
The distribution of somatic mutations of *CRP*-286 SNP and *APC* in tumors. The *CRP*-286 SNP mutations tend to co-occur with *APC* mutations in colon (A) (Fisher’s exact test, two-tailed, *p* = 0.47) and rectal cancers (B) (Fisher’s exact test, two-tailed, *p* = 0.04). Each rectangle represents one tumor sample with grey color denotes wild type status. The bar graphs on the right indicate the percentages of patients carrying APC mutations in two patient groups with or without CRP-286 mutations.

By contrast, a two-fold enrichment of mutant *APC* were observed in colon tumors with the concurrent *CRP*-286 SNP mutations (n = 38; Table 5 and Figure 3A). However, such a correlation did not reach the statistical significance probably owing to the limited sample size that we could obtain. We thus further examined 67 tumor/normal sample pairs of rectal cancer (Table 6 and Figure 3B), which is very similar to colon cancer in both the cell type origin and genomic alterations [25] showing high incidence of both *APC* [25] and the *CRP*-286 SNP mutations [19]. Indeed, the co-occurrence of these two mutations in this sample set became more evident (odds ratio: 5.56, 95% CI: 1.17–26.36) and significant (*p* = 0.04). These results thus suggest that CRP and APC may cooperate in overlapping pathways during the development of colorectal cancer.

**Table 5:** Clinicopathologic features of 38 colon cancer patients whose tumor/normal sample pairs were examined for *APC* mutations.

		Numberof Patients (%)	Number of patientswith *CRP*-286 mutation (%)	*p* *	Number ofpatients with*PC* mutation(%)	*p* *
**N**		38				
**Age**	<56 y	19 (50)	11 (61)	0.33	2 (20)	0.06
	≥56 y	19 (50)	7 (39)		8 (80)	
**Gender**	Female	13 (34)	7 (39)	0.73	3 (30)	1
	Male	25 (66)	11 (61)		7 (70)	
**Tumor Stage**	0–2	23 (61)	11 (61)	1	8 (80)	0.26
	3–4	15 (39)	7 (39)		2 (20)	
**Chemotherapy** **status**	Naïve	31 (82)	14 (78)	0.69	7 (70)	0.35
	Priortreatment	7 (18)	4 (22)		3 (30)	

*Fisher’s exact test, two-tailed.

**Table 6:** Clinicopathologic features of 67 rectal cancer patients whose tumor/normal sample pairs were examined for *APC* mutations.

		Number ofPatients (%)	Number of patientswith*CRP*-286mutation (%)	*p* *	Number ofpatients with*APC* mutation(%)	*p* *
**N**		67				
**Age**	<58 y	31 (46)	3 (37.5)	0.72	7 (54)	0.56
	≥58 y	36 (54)	5 (62.5)		6 (46)	
**Gender**	Female	23 (34)	4 (50)	0.43	7 (54)	0.12
	Male	44 (66)	4 (50)		6 (46)	
**Tumor Stage**	0–2	32 (48)	4 (50)	1	4 (31)	0.22
	3–4	35 (52)	4 (50)		9 (69)	
**Chemotherapy** **status**	Naïve	50 (75)	4 (50)	0.19	10 (77)	1
	Prior treatment	17 (25)	4 (50)		3 (23)	

*Fisher’s exact test, two-tailed.

## Discussion

The *in vitro* activities of CRP [3], [4], [6],[11],[13],[14], including the recognition of endogenous or exogenous danger signals, regulation of complement activation, induction of proinflammatory cell responses, lead to the idea that CRP may function as a soluble pattern recognition receptor in the innate immunity and host defense [6], [7]. However, the lack of consistent support by research on animal models [26]–[34], human subjects [35], [36] and genetic epidemiology [15], [20], [21], [37] makes it uncertain whether CRP plays any significant role in chronic inflammation *in vivo* or simply represents a nonspecific marker as hinted by its acute phase expression pattern. In this regard, the identification of the highly recurrent *CRP*-286 SNP mutations in multiple types of human cancer [19] provides a compelling evidence that this protein is a potential driver of tumorigenesis and a core component of the regulatory network of inflammation.

Promoter mutations in *TERT* [38], [39] and *CRP* [19] constitute the first examples that non-coding regulatory regions can also be targeted to promote tumorigenesis by modulating the expression instead of the activities of key genes. However, it is somewhat unique in case of *CRP* that the mutation occurs at a common SNP site. This raises the concern whether SNP sites are generally more vulnerable to genetic alterations, leading to the high incidence of passenger mutations. To address this concern, we genotyped 24 SNPs of 141 tumor/normal sample pairs. These SNPs are located on 9 distinct chromosomes, and consist of 3 SNPs of *CRP*, 2 promoter SNPs of inflammatory cytokines, 1 SNP of a non-coding gene, 18 SNPs with unknown association. Despite that, all of the SNP sites were found to be mutated in tumors with only low background frequency. Therefore, the highly recurrent mutation at the *CRP*-286 SNP site is most likely the result of the selection by cancer development, but not simply due to general properties associated with SNP site or genomic location. It is, however, still possible that the -286 mutation is just a consequence of tumorigenesis and further functional assays are required to clarify this point.

Nonetheless, it is intriguing that although the 4 examined *CRP* SNPs all affect the serum level of CRP, only the -286 SNP is targeted by tumorigenesis. This would suggest that the effects of the other 3 SNPs are secondary to the -286 SNP, which may in part be explained by the dependence of *CRP* expression on promoter CpG methylation, an essential epigenetic mechanism in gene silencing [40]. Indeed, we have recently shown that high CRP expression is correlated with low promoter methylation, and vice versa [19]. Of the 5 CpG motifs in *CRP* promoter, the evolutionarily conserved -286 CpG appears to be the key, particularly for extrahepatic cell types, in determining the basal level of *CRP* expression [19]. As the majority of the -286 mutations are C>A/T transitions that disrupt the methylation motif, it is conceivable that such genetic alterations will in turn contribute to switching on the promoter activity of *CRP* likely via lowering the inhibitory methylation signal and facilitating the binding of transcription factors to the underlying E-box sequence [41]. These may eventually allow the subsequent participation of distal regulatory elements containing the other *CRP* SNPs.

The high recurrence and pervasiveness of the *CRP*-286 SNP mutations in tumors suggest that locally produced CRP, instead of circulating CRP, drives the development of cancer. This paradox may be explained by the tight dependence of the actions of CRP on inflammatory microenvironments [3], [13], [14], [36]. Circulating CRP is produced by the liver as a pentamer primarily showing anti-inflammatory activities [7], [42], [43]. Besides hepatocytes, extrahepatic cells are also able to secrete CRP locally in response to inflammatory stimuli. Moreover, triggers enriched in inflammatory loci will induce prompt conformation changes in the pentameric CRP post its *in situ* production [44]–[49], to release the full potential in ligand binding [47], [48], [50], complement regulation [46], [48], [51]–[54] and stimulation of proinflammatory and angiogenic cell responses [48], [49], [55]–[61]. As such, the local abundance of CRP and its interactions with the stressful microenvironment should be more relevant to disease progression; while circulating CRP levels mainly mirror the underlying inflammatory status.

The dysregulation of Wnt signaling pathway is the most frequent event observed in colorectal cancer, which is usually manifested by inactivating mutations of *APC* or activating mutations of *β-catenin* [25]. One direct consequence of *APC* inactivation is the stabilization of *β-catenin* and the aberrant activation of the downstream target genes [62]. It is therefore of interest that *CRP* has been shown to be a target of *β-catenin* [63]. Moreover, our results reveal that the *CRP*-286 SNP mutations tend to co-occur with mutant *APC* in colon and rectal tumors. These would imply that the two secretory molecules, *i.e.* CRP and Wnt, may act in feed-back and cooperative manners to promote tumorigenesis, which deserves further investigations. Given the aberrantly activated Wnt signaling and highly induced *CRP* expression in tumors, topical targeting both molecules may be a potential option for colorectal cancer therapy.
